# Dietary diversity in primary schoolchildren of south-central Côte d’Ivoire and risk factors for non-communicable diseases

**DOI:** 10.1186/s12887-022-03684-6

**Published:** 2022-11-09

**Authors:** Sylvain G. Traoré, Kouadio B. Kouassi, Jean T. Coulibaly, Johanna Beckmann, Bomey C. Gba, Christin Lang, Kurt Z. Long, Daouda Dao, Markus Gerber, Nicole Probst-Hensch, Uwe Pühse, Jürg Utzinger, Bassirou Bonfoh

**Affiliations:** 1 Université Peleforo Gon Coulibaly, Korhogo, Côte d’Ivoire; 2 Centre Suisse de Recherches Scientifiques en Côte d’Ivoire, Abidjan 01, Côte d’Ivoire, 01 BP 1303 Abidjan, Côte d’Ivoire; 3 Université Nangui Abrogoua, Abidjan, Côte d’Ivoire; 4 Université Félix Houphouët-Boigny, Abidjan, Côte d’Ivoire; 5 Department of Sport, Exercise and Health, University of Basel, Basel, Switzerland; 6 Swiss Tropical and Public Health Institute, Allschwil, Switzerland; 7 University of Basel, Basel, Switzerland

**Keywords:** Anaemia, Glucose levels (HbA1c), High-density lipoprotein cholesterol (HDL-C), Low-density lipoprotein cholesterol (LDL-C), Malaria, Prediabetes, Overweight

## Abstract

**Background:**

A balanced nutrition is important for children’s physical and cognitive development; yet, remains a challenge in many parts of low- and middle-income countries (LMICs). Early detection of nutritional deficiency and metabolic syndrome in school-aged children is necessary to prevent non-communicable diseases (NCDs) in later life. This study aimed at obtaining baseline data on health, nutritional status, and metabolic markers of NCDs among primary schoolchildren in Côte d’Ivoire.

**Methods:**

A cross-sectional survey was conducted among 620 children from 8 public primary schools located in the south-central part of Côte d’Ivoire. Underweight and overweight were defined as a body mass index (BMI; kg/m2) < 5th and 85th up to 95th percentile for sex and age, respectively. Dietary diversity of children was calculated based on a 24-hour recall conducted with the primary caretaker according to the guideline of Food and Agriculture Organization. Anaemia, malaria, low-density lipoprotein cholesterol (LDL-C), high-density lipoprotein cholesterol (HDL-C), and blood glucose levels (HbA1c) were assessed, using capillary blood samples. Logistic models were performed to identify risk factors associated with overweight, HDL-C, LDL-C, and HbA1c.

**Results:**

Among the 620 children (330 girls, 290 boys; M_age_ 8.0 (± 1.7) years), 530 children attended school in a semi-urban and 90 in a rural area. Around 60% of children had a medium dietary diversity score (DDS). Children in peri-urban areas consumed more cereals (80.2% vs. 63.3%, p < 0.05). Most children were normal weight (n = 496), whereas 3.9% of children classified as prediabetic, 5% were underweight, and 15% overweight. LDL-C and HDL-C levels of children were associated with age, high DDS, and moderate anaemia. A significant association was found between prediabetes and malaria infection, as well as medium and high DDS. Overweight was associated with malaria infection and moderate anaemia.

**Conclusion:**

Overweight, prediabetes, low HDL-C, malaria, and anaemia are the main concerns of children’s health in Taabo. Our findings highlight interactions between infectious diseases, particularly malaria, and NCD risk factors. Monitoring NCD risk and infectious disease comorbidity in LMIC paediatric populations simultaneously is essential to better understand the dual diseases burden and apply early prevention measures.

**Supplementary Information:**

The online version contains supplementary material available at 10.1186/s12887-022-03684-6.

## Background

Throughout the world, school-aged children suffer from various infectious and preventable non-communicable diseases (NCDs) [1]. Current epidemiological data indicate that overweight, obesity, and diet-related NCDs are growing rapidly, particularly in low-to-middle income countries (LMICs) [2], while prevalence of stunting in children (impaired growth from poor nutrition) has declined markedly over the past 20 years [3]. Yet, 65 million children, with the majority living in low-to-middle income countries (LMICs), still suffer from chronic undernutrition due to inadequate food intake, infectious diseases, or both [4]. In fact, approximately 60 million children attend school hungry with about 40% residing in Africa [5]. Hence, underweight, as well as risk factors for NCDs and obesity are important health concerns for school-aged children living in LMICs [6].

An adequate nutritional diet is important in providing nutrients such as proteins, fats, vitamins, and minerals that are essential to the development, health, and wellbeing of individuals [7]. Optimum nutrition plays a pivotal role in the brain development of children [8] and is essential for a normal growth, and physical and cognitive development of infants and children. A balanced diet optimizes health, development, and performance at school [9]. Prior to puberty, school-aged children have a slow and steady growth pattern, so they need a wide variety of nutritious foods, which must be gradually adapted in portion size and quantity to meet their increasing energy requirements. The “zero hunger” Sustainable Development Goal (SDG) is not only to “eradicate hunger”, but also to “ensure that everyone has access all year round to healthy, nutritious and sufficient food” (SDG target 2.1) and to “put an end to all forms of malnutrition” (SDG target 2.2) [3]. Yet, the relationship between food and health is complex. We all require food to live and perform, however, too little food, too much food, or the wrong type of food can have negative consequences for health [10]. Diseases, as well as disability and weakness are closely related to nutritional deficiencies [7]. While a large body of research has studied undernutrition of children from various LMICs, data on obesity in rural parts are scarce, including West Africa. In 2011, a survey on childhood obesity in Senegal reported a prevalence of 9.3% [11], whereas a study conducted in Abidjan, the economic capital of Côte d’Ivoire, reported a prevalence of obesity among schoolchildren of 5% in 2012 [12]. Given the rapid lifestyle and nutrition transition that has also reached rural areas in Côte d’Ivoire, there is a high need to assess current NCD risk status among school-aged children. At the same time, children in rural Côte d’Ivoire still suffer from infectious diseases, such as malaria, constituting a significant dual disease burden. In fact, NCDs and infectious diseases share common features, such as increased mortality and morbidity and overlapping high-risk populations. There are also notable direct interactions between certain NCD risk factors and infectious diseases [13]. For instance, malaria is highly endemic in the Taabo area, which is located in the south-central part of Côte d’Ivoire, with the highest malaria prevalence among infants and young children (aged ≤ 9 years) [14]. There is evidence that malaria infection was associated with diabetes [15]. Malaria may increase the incidence and severity of malnutrition, while malnutrition may increase the risk of malaria [16]. Yet, the relationship between malaria, nutritional status, and NCD risk factors is complex. Optimal nutrition is critical to provide high immunity against environmental pathogens [17]. Diverse food is generally associated with good health of children and decreases malnutrition in children [18]. Nutritional-related behaviors are the most significant risk factors for NCDs, which are established during childhood and adolescence and often persist into adulthood [19]. Therefore, addressing risk factors of NCDs early in life can prevent or delay the onset of diabetes, obesity, and cardiovascular diseases [20].

In the Taabo area, previous research has mainly focused on malaria, anemia, and neglected tropical diseases (NTDs) [14, 21–25]. For example, a prior study pertaining to the etiology of anemia revealed significant associations between anemia and *Plasmodium falciparum* for infants, inflammation for school-aged children, and iron deficiency for both school-aged children and women [24]. Only few studies have been conducted in the Taabo area regarding NCDs, and none of these have been conducted in children [15, 26, 27]. Yet, given the above mentioned background, children and adolescents should be prioritized as target groups for preventing NCDs [28]. Moreover, studying NCD risk and malaria comorbidity simultaneously in school-aged children living in the Taabo area may provide important insights into the prevention and treatment of the double disease burden.

Therefore, the purpose of the present study was three-fold. First, to assess the dietary diversity among primary schoolchildren living in the Taabo area, and to identify differences based on sex and urbanization (semi-urban Taabo-Cité vs. rural Taabo-Village). Second, to describe the health status of children, putting particular emphasis on weight status, prediabetes (as assessed via glycated haemoglobin; HbA1c), high-density-lipoprotein cholesterol categories (HDL-C), low-density-lipoprotein cholesterol categories (LDL-C), malaria, and anaemia, stratified by sex and place of residence. Third, to examine whether risk factors for NCDs (overweight, prediabetes, low HDL-C, and high LDL-C levels) are associated with dietary diversity, after adjusting for children’s sex, age, place of residence, malaria, and anaemia.

## Methods

### Study design and location

The data presented here stem from the baseline assessment conducted within the frame of the *KaziAfya* cluster-randomized controlled trial [29]. Schoolchildren who underwent this cross-sectional study are involved in a multi-country intervention study examining the effects of a school-based health promotion programme focusing on physical activity and multi-micronutrient supplementation intervention on children’s growth, health, and wellbeing in Côte d’Ivoire, South Africa, and Tanzania.

The baseline data assessment took place in October 2018 in eight public primary schools in Taabo-Cité and Taabo-Village, which belong to the Taabo Primary Education Inspectorate. Taabo is located in the region of Agneby-Tiassa, in the south-central part of Côte d’Ivoire, approximately 150 km North-west of the economic capital Abidjan. Taabo was chosen as a study site because it harbours a demographic and health surveillance system (HDSS) that includes the small town of Taabo-Cité, 13 main villages (including Taabo-Village), and more than 100 hamlets. The total population covered by the Taabo HDSS in 2018 was approximately 42,500 inhabitants from 6,707 households. The Taabo HDSS serves as a platform for research, and evaluation of interventions on people’s health and wellbeing [30].

### Sample size calculation

To be able to detect at least a small effect in the intervention trial and to consider the weight status of the children (underweight, normal weight, or overweight/obese), power calculations indicated that a total sample of 1,096 children was needed (calculations based on G*power 3.1 with f = 0.10, alpha error probability = 0.05, power = 0.80, number of groups = 12, and number of measurements = 3). Assuming a yearly dropout-rate of 20%, the targeted sample size was 1,315 children. Sample size details are described elsewhere [29].

### Participants and procedure

Overall, 1,378 children from grades 1–4 (aged 5–12 years) participated in the baseline data assessment of the *KaziAfya* trial in Côte d’Ivoire. The children inclusion criteria were (i) attend grades 1–4 in one of the 8 selected schools with a maximum age of 12 years; (ii) have a written informed consent signed by the parent/guardian; (iii) do not participate in any other study; and (iv) do not suffer from any known chronic disease.

### Data collection

#### Dietary diversity score (DDS) information collection

DDS is used as a measure of dietary diversity. DDS is calculated by the sum of the number of food groups in a given reference of time based on a 24-hour recall. Parents/guardians were interview on their child’s consumption of 12 food groups within the past 24 h according to guidelines put forth by the Food and Agriculture Organization (FAO) for measuring household and individual dietary diversity [31]. In the presence of their children, parents/guardians were asked to report each food item (independently of quantity) that their child consumed in the previous 24 h. The recall period of 24 h corresponds with the recall period used in many previous dietary diversity studies [32, 33]. Overall, 149 food items considering foods in the locality were assessed. To calculate the DDS, the reported food items were categorized into 12 food groups (Table 1). Consumption of each food group was transformed into a dummy variable (1 = yes, 0 = no) to indicate whether or not items from a particular group were consumed. Based on the data from the 24-hour recall of the children’s parents/guardians, an aggregated DDS was created by summing the number of food groups reported by each parent/guardian for the household of his/her child (possible range from 0 to 12). The DDS was then divided into three categories for independent analysis. A DDS of < 4 food groups was regarded as low or inadequate dietary diversity, medium DDS comprised 4–6 food groups, and a high DDS consisted of 7–12 food groups [34–36].

**Table 1:** Composition of 12 food groups based on which a dietary diversity score (DDS) was calculated in the Taabo area, south-central Côte d’Ivoire in 2018

No.	Food group	Composition
1	Cereals	Rice and fonio
2	Legumes	Groundnut and beans
3	Roots and tubers	Yam and cassava
4	Vegetables	Garlic and eggplant
5	Fruits	Pineapple and avocado
6	Milk	Cow milk and ice cream
7	Eggs	Fresh eggs and mayonnaise
8	Meat	Chicken and beef
9	Fats	Oil and butter
10	Fish	Fresh fish caught from Lake Taabo
11	Sweets	Honey and candies
12	Beverages	Citronella

#### Anthropometric measurements

Body weight was measured and assessed with a wireless body composition monitor (BC-500; Tanita Corp.; Tokyo, Japan). The participants were instructed to morning fast on the day of data assessment, and to void their bladder immediately before data collection. With the shoes off, each child stood against a stadiometer with his/her back erect and shoulders relaxed. Body weight was measured to the nearest 0.1 kg. Body height was taken to the nearest 0.5 cm. Children’s body mass index (BMI) was converted to age- and sex-specific percentiles, according to recommendations of an Expert Committee Regarding the Prevention, Assessment, and Treatment of Child and Adolescent Overweight and Obesity [37–39]. Underweight was defined as an BMI < 5th, normal weight, from 5th to 85th, overweight from 85th to 95th and obese > 95th percentile for sex and age [40].

#### Biochemical data collection

Children underwent a clinical examination for the assessment of anemia, malaria, blood lipid profiles (HDL-C and LDL-C), and the detection of diabetes or prediabetes by analysis of capillary blood samples.

For the detection of anemia, the hemoglobin (Hb) concentration was measured once with a HemoCue® Hb 301 system (Ängelholm, Sweden), according to the manufacturer’s instructions. According to WHO classification, children aged 5–11 years with Hb concentrations < 11.5 g/dl were considered as being anemic. Children with Hb concentrations between 11.0 and 11.4 g/dl were considered to have mild anemia, those with Hb concentrations between 8.0 and 10.9 g/dl were considered to have moderate anemia, and those with Hb concentrations < 8.0 g/dl were considered to have severe anemia [41].

Malaria was detected by using a rapid diagnostic *test (*ICT Diagnostics; Cape Town, South Africa) for *P. falciparum*.

For the assessment of blood lipid profiles (LDL-C and HDL-C), capillary samples for blood lipid were analyzed with the Affinion™ 2 point-of-care (POC) analyzer (Abbott; Wädenswil, Switzerland). One drop of blood was taken up by the test strip and read directly by the machine. POC values were available within 8 min. We used the upper limit of acceptable values of lipid profiles according to the Expert Panel of National Heart, Lung, and Blood Institute on Integrated Guidelines for Cardiovascular Health and Risk Reduction in Children and Adolescents [42, 43]. The upper limit of acceptable values of LDL were as follows: <2.8 mmol/l = acceptable, 2.8–3.3 mmol/l = borderline high, and ≥ 3.4 mmol/l = high. The cut-offs for HDL-C were as follows: <1.027 mmol/l = abnormal and > 1.027 mmol/l = normal [44].

For the detection of diabetes risk, glycated hemoglobin levels (HbA1c) were determined from capillary blood samples with the Afinion™ 2 POC analyzer (Abbott; Wädenswil, Switzerland). One drop of blood was taken up by the test strip and read directly by the machine. POC values were available within 4 min. The HbA1c level reflects the average plasma glucose concentration levels over the previous 8–12 weeks before measurement with no prior fasting required [45]. The presence of the impaired fasting glycaemia (IFG) or prediabetes was detected as HbA1c between 5.7% and 6.5% and the presence of diabetes was detected as HbA1c of 6.5% (48.0 mmol/mol or higher) according to WHO diagnostic thresholds [46, 47]. The state of prediabetic is an intermediary condition of carbohydrate metabolism disorder before having diabetes II, which is reversible [48].

### Statistical analysis

Data were double entered and validated using EpiData (version 4.6.0.2) and merged into a single database. Descriptive statistics regarding dietary diversity and health outcomes are presented as arithmetic mean (M), standard deviation (SD), absolute frequency (N), and relative frequency (%), separately for the total sample, boys and girls, and children living in Taabo-Cité and Taabo-Village. Descriptive statistics and χ^2^ tests were assessed with SPSS (version 20, IBM; Armonk, USA). Chi-square (χ^2^) tests were performed to compare dietary diversity and health outcomes of boys and girls, and children living in Taabo-Cité and Taabo-Village. Statistical significance was defined at a p-value < 0.05. Logistic regression analysis, adjusting for clustering effects, was used to test for differences between living area (Taabo-Cité vs. Taabo-Village). To account for the disproportionate stratified sampling strategy from Taabo HDSS, all analyses were weighted using fixed weights derived from the inverse probabilities of selection.

We used a 2-sample test for equality of proportions to compare proportions of children with anaemia and malaria. For χ^2^ tests and logistic regression analyses, the health outcome variables, were categorized into binary variables: weight status (1 = overweight, 0 = no overweight), prediabetes (1 = prediabetes, 0 = no diabetes), HDL-C (1 = abnormal, 0 = normal), and LDL-C (1 = abnormal, 0 = normal), while sex, age, place of residency, malaria, anaemia, and dietary diversity were used as fixed variables in logistic regression models. Of note, four separate regression analyses were carried out based, respectively, on weight status, HbA1c, HDL-C, and LDL-C as outcome variables. Variables included into the best fitted model of each regression were selected using a stepwise selection and AIC criteria. We used R version 4.0.3 (2020 The R Foundation for Statistical Computing) for logistic regression analysis.

## Results

### Sample characteristics

**Fig. 1:**
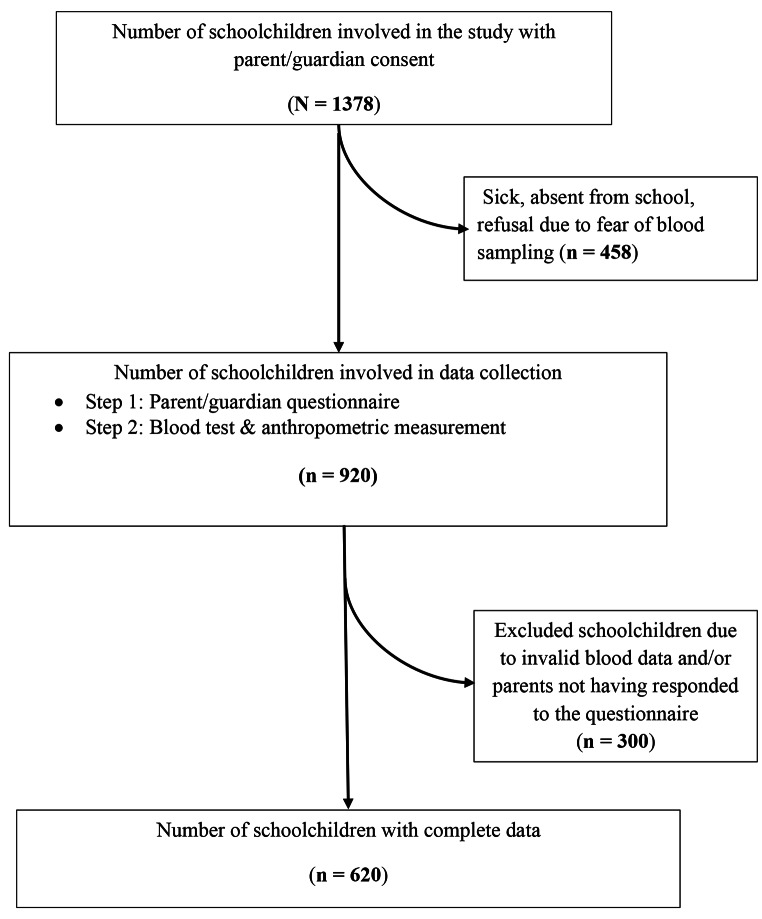
Study flow chart

A total of 1,378 children from selected schools consented to participate. Children’s age ranged from 5 to 12 years with an average of 8.0 years (SD = 1.7 years). Complete data for all variables relevant for the present paper were obtained from 620 children (330 girls and 290 boys). In most cases, missing data were caused by the absence of children during data collection, refusal of blood assessment, and invalid blood measures (Fig. 1). Of the 620 children with complete data, 90 children (14.5%) were from rural areas (Taabo-Village) and 530 (85.5%) from peri-urban areas (Taabo-Cité).

.

### Dietary intake of children

As shown in Table 2, the most frequently consumed food groups among children were vegetables (84.2% of girls *versus* 85.2% of boys), fish (81.8% of girls *versus* 82.1% of boys), beverages (82.1% of girls *versus* 75.9% of boys), and cereals (75.2% of girls *versus* 80.7% of boys).

Children in the semi-urban Taabo-Cité were consuming more cereals compared to those living in rural Taabo-Village (p < 0.05). Children from semi-urban Taabo-Cité were consuming less beverages (78.1% vs. 85.6%), fish (80.1% vs. 87.8%) and vegetables (84.0% vs. 88.9) compared to children from rural Taabo-Village. However, no statistically significant difference was observed (p > 0.05).

### Dietary diversity score

Table 3 shows DDS among children, stratified by sex and place of residence. The most frequent DDS was the medium. A comparison between boys and girls showed that a medium DDS was observed in 194 girls (58.8%) and 170 boys (58.6%) with no statistical difference.

A comparison between children according to their place of residence showed that a medium DDS was found in 56.8% of children from Taabo-Cité and 70.0% from Taabo-Village. No statistically significant difference was observed in DDS between children living in Taabo-Cité and Taabo-Village.

**Table 2:** Food group consumption among children according to the sex and place of residence

Food groups	Total	Female	Male	p-value	Taabo-Cité	Taabo-Village	p-value
	**N**	**n (%)**	**n (%)**		**n (%)**	**n (%)**	
	620	330 (53.2)	290 (46.8)		530 (85.5)	90 (14.5)	
Roots and tubers	332	184 (55.8)	148 (51.0)	0.273	263 (49.6)	69 (76.7)	< 0.001*
Cereals	482	248 (75.2)	234 (80.7)	0.119	425 (80.2)	57 (63.3)	< 0.001*
Vegetables	525	278 (84.2)	247 (85.2)	0.834	445 (84.0)	80 (88.9)	0.298
Fruits	312	165 (50.0)	147 (50.7)	0.928	267 (50.4)	45 (50.0)	1.000
Milk	51	33 (10.0)	18 (6.2)	0.117	49 (9.2)	2 (2.2)	0.042*
Eggs	2	1 (0.3)	1 (0.3)	1.000	2 (0.3)	0 (0.0)	1.000
Legumes	159	90 (27.3)	69 (23.8)	0.369	138 (26.0)	21 (23.3)	0.680
Meat	102	53 (16.1)	49 (16.9)	0.864	93 (17.5)	9 (10.0)	0.103
Fish	508	270 (81.8)	238 (82.1)	1.000	429 (80.1)	79 (87.8)	0.159
Fats	312	164 (49.7)	148 (51.0)	0.801	273 (51.5)	39 (43.3)	0.187
Sweets	138	70 (21.2)	68 (23.4)	0.568	126 (23.8)	12 (13.3)	0.039*
Beverages	491	271 (82.1)	220 (75.9)	0.069	414 (78.1)	77 (85.6)	0.142

p < 0.05: Statistical significance

**Table 3:** Dietary diversity, by children’s sex and place of residence

Category	Total	Female	Male	p-value	Taabo-Cité	Taabo-Village	p-value
	**N**	**n (%)**	**n (%)**		**n (%)**	**n (%)**	
	620	330 (53.2)	290 (46.8)		530 (85.5)	90 (14.5)	
DDS							
Low	64	33 (10.0)	31 (10.7)	0.958	58 (10.9)	6 (6.7)	0.060
Medium	364	194 (58.8)	170 (58.6)		301 (56.8)	63 (70.0)	
High	192	103 (31.2)	89 (30..7)		171 (32.3)	21 (23.3)	

DDS = dietary diversity score

### Health status of children

#### Underweight and overweight

Table 4 shows that among the 620 children, 31 (5%) were underweight and 93 (15%) were overweight. Regarding weight status, we did not observe any statistical significant difference in children between sex (male and female) and place of residence (Taabo-Cité and Taabo-Village).

#### Blood lipids and glycated haemoglobin

Table 4 shows that 90.0% of girls and 93.1% of boys had a normal LDL-C level (< 2.8 mmol/l). With regard to sex, 10.0% of girls and 6.9% of boys presented with abnormal LDL-C levels (≥ 2.8 mmol/l). With regard to place of residence, no statistically significant difference was found for LDL**-**C level between children living in Taabo-Cité and Taabo-Village.

Concerning HDL**-**C level, among the 620 children, 324 (52.3%) had an abnormally low rate of HDL-C (< 1.0 mmol/l), with 50.9% of girls and 53.8% of boys falling below this cut-off. A statistically significant difference was found for HDL**-**C level between children in Taabo-Cité and Taabo-Village (Table 4).

Table 4 further shows that among the 620 children, 24 (3.9%) were classified as prediabetic. In total, 2.7% of girls and 5.2% of boys had a HbA1c level set between 5.7% and 6.5% (Table 4). Neither for sex nor for place of residence were significantly associated with HbA1c level. The percentage of prediabetic children was 4.3% in Taabo-Cité and 1.1% in Taabo-Village.

### Malaria and anaemia

The mean Hb level was 11.9 g/dl in malaria-free children and 11.3 g/dl in children with malaria. Among children with malaria , 55.6% (n = 174) were anemic vs. 30.3.0% (n = 93) (χ^2^ = 40 .45, p < 0.0001). Children suffering both of malaria and anemia were mainly found in Taabo-Cité (81.0%, n = 141) rather than Taabo-Village (19.0%, n = 33) (χ^2^ = 131.60, p < 0.001).

Table 4 shows that half (50.5%) of the children had malaria. Statistically significant difference was found for malaria between children in Taabo-Cité and Taabo-Village.

**Table 4:** Health parameters, by sex and place of residence

Category	Total	Female	Male		Taabo-Cité	Taabo-Village	
	**N**	**n (%)**	**n (%)**	**p-value**	**n (%)**	**n (%)**	**p-value**
	620	330 (53.2)	290 (46.8)		530 (85.5)	90 (14.5)	
**Weight status**							
Underweight < 5th	31	20 (6.1)	11 (3.8)	0.285	26 (4.9)	5 (5.6)	0.213
Normal weight [5th ; 85th [	496	257 (77.9)	239 (82.4)		419 (79.1)	77 (85.6)	
Overweight [85th ; 95th [	93	53 (16.1)	40 (13.8)		85 (16.0)	8 (8.9)	
**Low-density-lipoprotein cholesterol**							
Normal (< 2.8 mmol/l)	567	297 (90.0)	270 (93.1)	0.217	483 (91.1)	84 (93.3)	0.626
Abnormal (≥ 2.8 mmol/l)	53	33 (10.0)	20 (6.9)		47 (8.9)	6 (6.7)	
**High-density-lipoprotein cholesterol**							
Abnormally low (< 1.027 mmol/l)	324	168 (50.9)	156 (53.8)	0.524	264 (49.8)	60 (66.7)	0.004*
Normal (≥ 1.027 mmol/l)	296	162 (49.1)	134 (46.2)		266 (50.2)	30 (33.3)	
**Glycated haemoglobin**							
Normal (< 5.7%)	596	321 (97.3)	275 (94.8)	0.172	507 (95.7)	89 (98.9)	0.241
Prediabetic (5.7 to 6.5%)	24	9 (2.7)	15 (5.2)		23 (4.3)	1 (1.1)	
**Haemoglobin**							
No	353	200 (60.6)	153 (52.8)	0.048*	310 (58.5)	43 (47.8)	0.161
Mild anaemia	115	59 (17.9)	56 (19.3)		92 (17.4)	23 (25.6)	
Moderate aanemia	149	68 (20.6)	81 (27.9)		125 (23.6)	24 (26.7)	
Severe anaemia	3	3 (0.9)	0 (0.0)		3 (0.6)	0 (0.0)	
**Malaria**							
Not infected	307	162 (49.1)	145 (50.0)	0.884	272 (51.3)	35 (38.9)	0.039*
Infected	313	168 (50.9)	145 (50.0)		258 (48.7)	55 (61.1)	

* p < 0.05: Statistically significant

### Risk factors associated with prediabetes, blood lipids level, and overweight

Table 5 shows that the LDL level of children was statistically significantly associated with sex, age, high DDS, and moderate anaemia. Boys had a lower odd of LDL-C than girls (odds ratio (OR) = 0.69, (95% confidence interval (CI): 0.68–0.70). Compared to children with severe anaemia, those with moderate anaemia had 0.53 times decreased odds of having abnormal LDL-C (OR = 0.53 [95% CI: 0.42–0.66]). Increasing age was associated with lower odds of having abnormal LDL-C levels (OR = 0.81 [95% CI: 0.78–0.84]). Low DDS was less prevalent in children with abnormal LDL-C (high level) (OR = 1.54 [95% CI: 1.07–2.21]) than high DDS.

Children’s HDL-C levels were statistically significantly associated with malaria infection status, age, high DDS, and moderate anemia. Increasing age was associated with higher odds of having abnormal HDL-C levels (OR = 1.19 [95% CI: 1.14–1.23]). Low DDS was more prevalent in children with abnormal HDL-C (low level) (OR = 0.74 [95% CI: 0.56–0.97]) than high DDS. Compared to children with severe anaemia, those with moderate anaemia had 3.33 times increased odds of having abnormal HDL-C (OR = 3.33 [95% CI: 2.97–3.73]). Compared to children without malaria, those with malaria had 3.15 times increased odds of having abnormal HDL-C (OR = 3.15 [95% CI: 1.94–5.11]) (Table 5).

Prediabetes was statistically significantly associated with malaria infection status, medium and high DDS. The odds of being prediabetic was 1.94 [95% CI: 1.15–3.27] times (p = 0.013) and 2.64 [95% CI: 1.22–5.70] times (p = 0.014) higher in children with medium and high DDS, respectively, if compared to children with low DDS (Table 5).

Overweight was statistically significantly associated with malaria infection status and moderate anaemia. Compared to children with severe anaemia, those with moderate anemia had 5.08 times increased odds of being overweight (OR = 5.08 [95% CI: 1.75–14.75]). Compared to children without malaria, those with malaria had 0.75 times decreased odds of being overweight (OR = 0.75 [95% CI: 0.68–0.83]) (Table 5).

**Table 5:** Risk factors associated with prediabetes, abnormal blood lipid level and overweight

	Estimate	Std. error	t-value	Adjusted OR [95%CI]	p-value	
**Abnormal low-density lipoprotein cholesterol**						
Age	-0.211	0.018	-11.524	0.81 [0.78–0.84]	< 0.001	
Sex: Male	-0.370	0.000	913.061	0.69 [0.68–0.70]	< 0.001	
DDS medium	0.429	0.428	1.003	1.54 [0.66–3.56]	0.316	
DDS high	0.428	0.186	2.303	1.56 [1.07–2.21]	0.021	
Mild anaemia	-0.083	0.973	-0.086	0.92 [0.14–6.20]	0.932	
Moderate anaemia	-0.643	0.114	-5.666	0.53 [0.42–0.66]	<0.001	
Malaria: infected	-0.283	0.213	-1.326	0.75 [0.50–1.15]	0.185	
**Abnormal high-density lipoprotein-cholesterol**						
Age	0.172	0.018	9.610	1.19 [1.15–1.23]	< 0.001	
Sex: Male	-0.095	0.350	-0.272	0.91 [0.46–1.87]	0.786	
DDS medium	-0.059	0.361	-0.162	0.94 [0.47–1.91]	0.871	
DDS high	-0.305	0.139	-2.192	0.74 [0.56–0.97]	0.028	
Mild anaemia	0.693	0.512	1.354	2.00 [0.73–5.46]	0.176	
Moderate anaemia	1.202	0.059	20.453	3.33 [2.97–3.73]	< 0.001	
Malaria: infected	1.146	0.248	4.629	3.15 [1.94–5.11]	< 0.001	
**Prediabetes**						
Age	-0.029	0.020	-1.411	0.97 [0.93–1.01]	0.158	
Sex: Male	0.509	0.381	1.334	1.66 [0.79–3.51]	0.182	
DDS medium	0.662	0.267	2.474	1.94 [1.15–3.27]	0.013	
DDS high	0.969	0.394	2.463	2.64 [1.22–5.70]	0.014	
Mild anaemia	-0.967	0.622	-1.554	0.38 [0.11–1.29]	0.120	
Moderate anaemia	0.150	0.492	0.304	1.16 [0.44–3.05]	0.761	
Malaria: infected	-2.145	0.234	-9.179	0.12 [0.07–0.19]	< 0.001	
**Overweight**						
Age	0.227	0.174	1.300	1.25 [0.89–1.77]	0.193	
Sex: Male	0.321	0.193	1.658	1.38 [0.94–2.01]	0.097	
DDS medium	0.297	1.279	0.232	1.35 [0.11–16.52]	0.816	
DDS high	-0.434	0.897	-0.484	0.65 [0.11–3.76]	0.629	
Mild anaemia	-0.095	1.004	-0.095	0.91 [0.13–6.51]	0.925	
Moderate anaemia	1.625	0.544	2.986	5.08 [1.75–14.75]	0.003	
Malaria: infected	-0.286	0.050	-5.755	0.75 [0.68–0.83]	< 0.001	

Statistical tests were conducted with logistic regression, adjusted for clustering, at living area and weighted to avoid oversampling of children living in peri-urban area. * p < 0.05: Statistical significance. Reference are female for sex, DDS low for DDS, severe anemia for anemia and Uninfected for malaria

## Discussion

The purpose of the present study was to shed light on risk factors for NCDs among schoolchildren living in a low-income country, namely Côte d’Ivoire. Hence, in the Taabo area in the south-central part of the country, diet and health status of schoolchildren were examined, which are potenital risk factors for developing NCDs in later life. A key finding was that the majority of children were normal weight, yet, 5% were underweight and 15% overweight. Prediabetes was diagnosed in 3.9% of the children, which was significantly associated with malaria infection, as well as medium and high dietary diversity.

The most consumed food groups of 5- to 12-year-old schoolchildren in the Taabo area were vegetables, fish, beverages, and cereals. The consumption was not related to children’ sex, but place of residence. Children in semi-urban Taabo-Cité consumed more cereals than their rural counterparts. These foods are probably commonly consumed by parents and schoolchildren in their households due to their availability. We conjecture that there is sufficient fish as source of protein, which is explained by the close proximity of the Bandama River and the man-made Lake Taabo. One of the most consumed food groups by schoolchildren (e.g., cereals) in our study was also consumed by the majority of the children in a study done in 2019 by Ayogu [49] in children aged 6–15 years in Ede-Oballa, a suburban area located in South Nigeria. Indeed, starchy staples (cereals and starchy roots, tubers, and fruits), fats and oil, legumes, nuts, and seeds were consumed by the majority of the Nigerian children.

Concerning the DDS of children, which is defined as the number of food groups consumed over a period of 24 h [50], around 60% of the children presented with a medium DDS. There is a range of possible reasons that may explain this finding. First, in the Taabo HDSS, approximately 72% of the population above the age of 6 years are illiterate [30]. Thus, parents may not be sufficiently educated to provide diverse and nutrient rich diet to their children who have very little influence on their food choices. As shown in prior research, children’s eating behavior is strongly influenced by parental food preferences and beliefs, food access and availability, child/parent interactions related to food, the behavior of other role models, presence of food allergies/intolerances, and the media [51, 52]. Second, families’ household socioeconomic status may provide a further explanation for the observed dietary diversity. In Côte d’Ivoire, most people are still living in rural settings (82%) [30]. Consequently, it is conceivable that many parents in our sample from the Taabo area did not have the economic means to provide a highly diverse diet to their children. In line with this observation, previous research has found that household expenditure is directly related to dietary diversity and food variety [53].

Similar to our study, a prior investigation in rural Nigerian schoolchildren reported that most children (78%) had medium DDS [49] and the proportion of children with a medium DDS (58.7%) was slightly lower in our study compared to the Nigerian sample. In the study implemented in the south-eastern part of Nigeria, a low DDS was observed in most of the households after the end of the planting season and just before harvest. In contrast, data assessment in the present study took place during the main harvest season. The difference observed in DDS between the two settings could be explained by the seasonality of some food items and particularly fruits. Indeed, in a study done in 2017 by Stevens et al. [54] in rural Bangladesh on the role of seasonality on diet and household food security, seasonality was found to be closely associated with dietary diversity.

A key finding related to health status of schoolchildren was that in the present population, 5% of children were underweight and 15% were overweight. Our results are in line with those obtained in a study carried out in Togo (urban areas of Lomé) [35], where the prevalence of overweight/obesity and underweight among schoolchildren were 7% and 18%, respectively. In our sample, overweight was statistically significantly associated with malaria infection status and a moderate anemia. Malnutrition was reported in Ghana [55], to be a fundamental factor contributing to malaria-associated morbidity and anemia, even if the latter is multifactorial. In a study done with adults in Sweden in 2015, obesity was strongly associated with severe malaria, both independently (adjusted OR: 5.58, 95% CI: 2.03–15.36) and in combination with an additional metabolic risk factor (hypertension, dyslipidaemia, or diabetes) (adjusted OR: 6.54, 95% CI: 1.87–22.88) [56]. Our findings are in contrast to those reported in a study carried out among children in 50 schools in Malawi [57]. Indeed, in this study, higher odds of *P. falciparum* infection were associated with younger age and being stunted.

Based on reference standards proposed by the Expert Panel of National Heart, Lung, and Blood Institute on Integrated Guidelines for Cardiovascular Health and Risk Reduction in Children and Adolescents [42, 43], more than 90% of children in our study had an acceptable level of LDL-C and only very few children (8.5%) were found with high LDL-C levels. In contrast, slightly more than half of the children presented with low HDL-C levels (with an especially high rate observed in Taabo-Village). HDL-C is an indicator of health status because it reduces blood clotting and prevents inflammation [58]. When HDL-C increases by 1 mg/dl, the risk of coronary artery decreases by 2–3% [59]. Our results concur with those of Lartey et al. [60], based on a study with children aged 9–15 years living in urban Ghana, where the percentage of children with abnormal values for LDL-C (high levels) and HDL-C level (low levels) amounted to 9% and 28%, respectively. Our study also showed that abnormal LDL-C and HDL-C levels of children were statistically significantly associated with age, high diet diversity, and moderate anaemia. Previous research showed that children with abnormal LDL-C and HDL-C levels are at risk for cardiovascular disease (CVD) in adulthood. For instance, in a study done in a Finnish population, higher CVD risk in adulthood was associated with higher BMI and levels of higher blood cholesterol level at the age of 12 years [61, 62]. Moreover, a 4-year longitudinal study among children aged 8–10 years from Japan showed that cholesterol levels are relatively stable across time, which indicates that abnormal levels might persist for several years [63]. Taken together, our findings suggest that abnormal HDL-C levels may indeed be an issue in children living in rural Côte d’Ivoire. However, the results should be interpreted with caution as no specific classification cut-offs exist for African schoolchildren.

In our sample of schoolchildren, approximately 4% were classified as prediabetic. These children are at increased risk of developing diabetes in later life [64]. The prevalence of prediabetic status found in our study is higher than the one (0.9%) reported in Yemen in a sample of 1,402 children aged 12–13 years [65]. In our study, prediabetes was statistically significantly associated with malaria infection status, medium and high DDS. The link of prediabetes and malaria can be explained by the fact that parasite growth was positively associated with blood glucose, HbA1c, BMI, fibrinogen, and triglycerides [66]. The study done in 2017 by Eze et al. [15] in Taabo among 979 adults, showed association between diabetes and parasite density.

Diabetes should be considered a major health issue in Côte d’Ivoire. According to the integrated strategic plan for the prevention and management of NCDs in Côte d’Ivoire from 2015 to 2019, age-standardized prevalence of diabetes in individuals aged 18 years and above was 10.7% in 2014 [67]. In 2017, at Taabo, in individuals aged between 18 and 87 years, the prevalence of fasting glucose-based prediabetes and diabetes were 45.8% and 3.6%, respectively. The prevalence of prediabetes and diabetes based on HbA1c- values were 2.7% and 0.7%, respectively [15]. Diabetes is not likely to be an important health problem in a sample where the majority of participants has normal weight. Whether the situation looks different in children living in urban settings needs further scientific inquiry. The findings of our study have direct public health implications and will guide future interventions in the HDSS with the ultimate goal to improve people’s health and wellbeing.

Our study has several limitations that are offered for consideration. First, we used parents/guardians’ recall of the child’s food intake in the previous 24 h. Using a single 24-hour recall period does not provide an indication of an individual’s habitual dietary behavior as there might be differences depending on the day of a week and according to seasons. Nevertheless, the recall period of 24 h is easier and less subject to recall error and it provides a valuable assessment of the diet at the population level and can be useful to monitor progress or target interventions [68]. Second, using a food item checklist, we did not obtain information on the amount/quantity of food consumed. Third, we did not collect data on children’s weight at birth, the educational attainment of their primary caregiver, and the socioeconomic status of parents. Such indicators might be relevant to better understand overweight [69] and prediabetes of children because being born underweight is in fact a risk factor for diabetes later in life.

Despite these shortcomings, our study sheds new light on the health status of children living in a low-income country and the risk for developing NCDs. The knowledge of children’s health status and the understanding of risk factor associated to their health status are important to follow them more closely for the planned implementation of a multi-country cluster randomized trial. As recommended by an expert panel on integrated guidelines for cardiovascular health and risk reduction in children and adolescents, attention should be paid to the cholesterol levels of children, because they are characterized by a considerable temporal stability and because they serve as a predictor of future CVDs in adulthood. Attention should also be paid to prediabetic children because children susceptible to abnormal carbohydrate metabolism have a markedly increased risk to getting diabetes-related CVDs later in life.

## Conclusion

We present one of the first studies to determine risk factors for NCDs among primary schoolchildren in a low-income country. Overweight, prediabetes, low HDL-C, malaria, and anaemia are the main concerns of children’s health in Taabo. Our findings highlight interactions between infectious diseases (ID) and NCD risk factors. Monitoring NCD risk and ID comorbidity in LMIC paediatric populations simultaneously is essential to better understand the dual diseases burden and apply early prevention measures.

## Electronic supplementary material

Below is the link to the electronic supplementary material.

Supplementary Material 1

## Data Availability

All data generated or analysed during this study are included in this published article [and its supplementary information files].

## Abbreviations

N: Absolute frequency
M: Arithmetic mean
BMI: Body mass index
CVD: Cardiovascular disease
CDC: Centers for Disease Control and Prevention
(χ^2^) test: Chi-square
HDSS: Demographic and health surveillance system
DDS: Dietary diversity score
FAO: Food and Agriculture Organization
FFQ: Food frequency questionnaire
Hba1c: Glycated haemoglobin
Hb: Haemoglobin
HDL-C: High-density lipoprotein cholesterol
IFG: Impaired fasting glycaemia
LDL-C: Low-density lipoprotein cholesterol
NTDs: Neglected tropical diseases
NCDs: Non-communicable diseases
POC: Point-of-care
%: Relative frequency
SD: Standard deviation
SDG: Sustainable Development Goal
WHO: World Health Organization
